# An Online Career Intervention for Promoting Chinese High School Students’ Career Readiness

**DOI:** 10.3389/fpsyg.2021.815076

**Published:** 2022-01-10

**Authors:** Shi Chen, Huaruo Chen, Hairong Ling, Xueying Gu

**Affiliations:** ^1^School of Education Science, Nanjing Normal University, Nanjing, China; ^2^Center for Research and Reform in Education, Johns Hopkins University, Baltimore, MD, United States

**Keywords:** online career intervention, career readiness, career decision-making difficulties, high school students, CIP theory

## Abstract

To assist Chinese high school students in improving their career readiness and tackling career decision-making difficulties, we designed a synchronous online career intervention based on the Cognitive Information Processing (CIP) theory during the Covid-19 pandemic. The online career intervention consisted of a series of career courses to develop high school students’ knowledge and skills in career planning, career assessments for exploring their vocational interests and academic self-concept, and a database providing basic information about university majors. To evaluate the intervention’s effectiveness, 957 10th grade students were recruited in the study, 601 participants (girls = 227, boys = 324) were randomly assigned to the experimental group (online career intervention), and 356 (girls = 159, boys = 197) participants were randomly assigned to the control group (no any career interventions). All participants completed a pre- and post-intervention assessment of their career maturity, vocational identity and career decision-making difficulties. Results indicated that the online intervention significantly increased high school students’ career readiness and reduced their career decision-making difficulties. The practical implications of this research for online career interventions directed at Chinese high school students are also discussed.

## Introduction

The rapid socio-economic development occurring in China over the last four decades has made the process of choosing a career increasingly challenging, especially under boundaryless careers (Kost et al., 2020). However, interventions to facilitate this process remain relatively undeveloped (Fan and Leong, 2016). In 2020, an important change to the Chinese university entrance examination came into effect. The admission process became more wide-ranging; whereas only test scores had previously been considered, the admission process became much more comprehensive, taking into account a range of measures including academic scores, extracurricular activities, physical and mental health, and moral behavior. In summary, the multiplicity of measures and choices under the new system requires high school students to engage in career planning at an early stage and demands greater rigor from schools in their approach to career counseling.

With the outbreak of various variants including Alpha, Beta and Omicron, the Covid-19 pandemic may not be able to stop in the short term. The Covid-19 pandemic has had an unprecedented effect on education across the world. To minimize the negative impacts of the pandemic on education teaching work, many countries and regions have made unified arrangements for a series of policies and measures (Xue et al., 2020; Mitescu-Manea et al., 2021; Ramot and Donitsa-Schmidt, 2021). In China, the Ministry of Education’s approach to the pandemic was summarized in the slogan “Suspension of Classrooms without Suspension of Studying” (The Ministry of Education, 2020), and has been achieved by moving many elements of formal education online. Online career interventions to improve students’ career choices, however, have remained largely absent from this process. We, therefore, set out to design and test an online career intervention aiming to help Chinese high school students during the Covid-19 pandemic.

## Literature Review

### Career Intervention

Several meta-analyses have provided a historical overview of the effectiveness of career interventions (Perdrix et al., 2012; Whiston and Rose, 2014), with the overall effect size of these found to be moderate. Nonetheless, individuals who benefited from interventions designed to boost their career decision-making abilities scored significantly higher on various measures than those who received no such assistance (Whiston et al., 2017). The development of Information Technology and the Internet has brought career educators with possibilities to evolve the teaching pedagogy and renovate the traditional face-to-face learning into a more engaging and effective approach (Pordelan et al., 2018). For instance, there have been numerous asynchronous and synchronous career courses on the Massive Open Online Course (MOOC) platform. More accessible than traditional career guidance centers, online career interventions allow students to access a wide variety of useful information (Pordelan and Hosseinian, 2021). One advantage of online career interventions is the availability of audiovisual materials including videos, slides, and cartoons, which can be used to explore students’ values, interests, and skills. They can also support the career decision-making process by gathering information about personal traits, education and possible vocations, facilitating matches to particular occupations (Nota et al., 2016). Online career interventions can be highly efficient and are likely to be used increasingly in the future (Dozier et al., 2015). The effectiveness of online career interventions is widely acknowledged (Nota et al., 2016; Teychenne et al., 2019; Pordelan et al., 2020) but has not yet been confirmed in the Chinese context, a fact supporting the need for the current study.

The gender of students may play a role in the effectiveness of career interventions. However, only a few studies have investigated this to date, with inconclusive results. Hechtlinger and Gati (2019) evaluated the effectiveness of a collective career intervention and found it impacted the dysfunctional career decision-making beliefs of female students more than those of their male counterparts. While Cassie and Chen (2012) found that male high school students’ career decision-making self-efficacy was positively affected by the support they received. Maree (2019) detected no gender difference in the effectiveness of high school career counseling, confirming the findings of other researches (Nota et al., 2016; Thul-Sigler and Colozzi, 2019). Finally, a study conducted in China concluded that high school girls retained more information about prospective careers whereas boys performed better in career decision-making self-efficacy following input (Gu et al., 2020). To help clarify the inconsistencies in these findings, the current study also investigated whether the effectiveness of the intervention varied by gender.

### A Cognitive Information Processing Theory-Based Career Intervention

Cognitive information processing (CIP) theory offers a promising framework combining theory, practice, and research which can be utilized to help individuals make appropriate decisions about their careers (Sampson et al., 2011). The first element of the framework was the information-processing pyramid, through which types of knowledge required in the career decision-making process can be identified. The information-processing pyramid consists of four essential components: (a) self-knowledge (e.g., interests, values, personalities, and abilities); (b) knowledge of options (e.g., information about educational pathways, the labor market, leisure options, and workplaces); (c) decision-making skills; and (d) executive processing (metacognition). Successful career decision-making should be based on deep understanding and the flexible integration of self-knowledge and knowledge of options (Osborn et al., 2020). The second is the CASVE cycle (Buzzetta et al., 2017), which describes the actions required in decision-making. The CASVE cycle is a decision-making model describing the process of career decision-making and problem-solving. The cycle consists of communication (identifying a career-related problem), analysis (clarifying problem components), synthesis (creating potential options), valuing (evaluating the options), and execution (making plans to implement the options) (Buzzetta et al., 2017). Communication, analysis, and synthesis take place early in the decision-making process; valuing and execution occur later.

Over 180 empirical studies have drawn on CIP theory, demonstrating its applicability to the study of career interventions (Wu, 2018). CIP theory has been successfully applied to a wide range of career services, including assessment, counseling, and other forms of support; its application in schools, career centers, and other organizations also indicates its relevance to the topic (Toh and Sampson, 2021). A CIP-based career course was designed for undergraduates which positively affected students’ career decision-making states and skills, their knowledge of the next steps to take, and their anxiety about careers (Osborn et al., 2020). Osborn and Belle (2019) described the potential applications of the CIP approach for improving the career readiness of juveniles. As previously mentioned, Chinese high school students typically encounter many difficulties in career decision-making (Xu et al., 2016; Gu et al., 2020): they are often unprepared, have limited knowledge of self and the outside world, and struggle to choose an appropriate university major as a result. The utility of CIP theory to addressing such issues informed the design of the online career intervention for high school students in the present study.

### Career Readiness and Career Decision-Making Difficulties

Career readiness refers to the readiness of an individual to engage in the career decision-making process and make a mature career decision (Hirschi and Läge, 2007). Career readiness helps young people set career goals, plan career paths, improve professional skills and knowledge and follow the designated path (Gates et al., 2018). For Chinese high school students, career readiness is an essential step in the transition from high school to university, as subjects are chosen with a view to enter and select a major at university (Gu et al., 2020). Career maturity and vocational identity are regarded as equivalent to career readiness (Johnson et al., 2014; Babarović et al., 2020): in this context, maturity refers to individuals’ readiness to make decisions about a future career (Savickas and Porfeli, 2011), and vocational identity refers to a component of career readiness; namely, establishing a commitment to career choices (Porfeli et al., 2011). Thus, career readiness could be measured by assessing career maturity and vocational identity. Hirschi and Läge (2008) designed a CIP-based career workshop to promote the career readiness of adolescents; to evaluate the effectiveness of the workshop, Career Maturity Inventory (Crites and Savickas, 1996) and Vocational Identity Scale (Holland et al., 1993) were selected to measure career readiness. Career Maturity Inventory (CMI)-Form C (Savickas and Porfeli, 2011) assesses four dimensions of the career decision-making process: career concern, career curiosity, career confidence and career consultation; the total derived from the sub-scores on career concern, career curiosity and career confidence represents the individual’s degree of career readiness, i.e., measures their preparedness to make appropriate vocational choices. Therefore, in the current study, the CMI- Form C and Vocational Identity Status Assessment (Porfeli et al., 2011) were applied to measure participants’ career readiness.

Many adolescents and young adults struggle to plan a future career path (Gati and Levin, 2014) and the difficulties they face must therefore be analyzed carefully in order to design more effective interventions. Career decision-making difficulties have been classified into three clusters (Gati et al., 1996; Duru et al., 2021): (1) lack of readiness, which exists before the career decision-making process begins; (2) lack of information; and (3) inconsistent information during the decision-making process. The three clusters are further subdivided into ten smaller categories: cluster 1 covers lack of motivation, general indecisiveness, and dysfunctional beliefs; cluster 2 concerns the absence of knowledge about the career decision-making process, of information about self and vocations, and the inability to obtain additional information; and the third cluster includes unreliable information and conflicts which may be internal or external. This model plays a major role in understanding the difficulties involved in the career decision-making process. To deal with career decision-making difficulties, students often seek professional advice from career counseling services. Career decision-making difficulties could be reduced *via* career interventions (Kulcsár et al., 2020). In order to assist students to eliminate their career decision-making difficulties, career counselors should identify the factors which link with these difficulties. Enhancing career decision-making self-efficacy was successful to reduce career decision-making difficulties (Lam and Santos, 2018). Emotion is essential in the career decision-making process, and bad emotions may result in more career decision-making difficulties (Santos et al., 2018). In general, it seems necessary for educators, counselors to help students handle their career decision-making difficulties, for the sake of making appropriate career choices and planning.

## Materials and Methods

### Purpose of the Study

Due to the Covid-19 pandemic, career educators were required to adopt online technology. Since no research has yet examined the effects of online career interventions in Chinese high schools, the current study’s primary purpose was to develop an online career intervention for Chinese high school students and investigate its effectiveness. Another contribution of the research was to examine the moderating variable of gender, due to the scarcity and inconsistencies of previous findings. In the present study, we made three main hypotheses:

Hypothesis 1: the online career intervention would increase high school students’ career readiness;

Hypothesis 2: the intervention would decrease students’ career decision-making difficulties;

Hypothesis 3: gender would be a significant moderator of the intervention effect.

### Participants

Year 10 participants were selected from two public high schools (12 classes from each school) in Jiangsu Province, China. We asked the participants if they wanted to participate, and we also get their parents’ agreement. The Ministry of Education’s recent initiative requires students to select any three subjects from among physics, chemistry, biology, history, politics, and geography; and future selection of university majors is based on the current selection of subjects. Once they confirm the three subjects, their choice is irrevocable, meaning that students must be well prepared to make their selection. However, due to school closures during the Covid-19 pandemic, most students struggled to plan their careers without any guidance or support from teachers. The online intervention was designed and delivered to address this issue.

Eight classes from School A and seven classes from School B were randomly assigned to the experimental group, with the remaining nine classes placed in the control group. One thousand and thirty-sixth participants completed the pre-test questionnaires (experimental group = 656; control group = 380); some participants dropped out of the study or missed one or more components of the intervention and some participants did not complete the post-test questionnaire. Overall, 957 students (Girls = 436, 45.6%, Boys = 521, 54.4%) with an overall mean age of 16.28 years old (SD = 0.42) participated. The experimental group consisted of 601 participants (*M*_age_ = 16.29 years, SD = 0.44; Girls = 227, 46.1%; Boys = 324, 53.9%), with 356 participants in the control group (*M*_age_ = 16.26 years, SD = 0.41; Girls = 159, 44.7%; Boys = 197, 55.3%). The analysis of variance across the experimental and control groups revealed no significant differences (*p* > 0.05) on all variables.

### Measures

Control Variables. A demographic questionnaire was developed to measure the demographic variables which included participants’ gender, age, birthplace (rural/urban), and parents’ educational levels, income levels, social status.

#### Career Readiness

We used the Career Maturity Inventory (CMI)–Form C (Savickas and Porfeli, 2011) and the Vocational Identity Status Assessment (Porfeli et al., 2011) to measure participants’ career readiness. The CMI-Form C contains twenty-four items: six items of career concern, career curiosity, career confidence and career consultation, respectively. Total scores of Career Concern sub-scale, Career Curiosity sub-scale and Career Confidence sub-scale represent the extent of readiness to make career choices. The CMI-Form C presents a choice of “Agree” or “Disagree” responses. “Agree” scores 0 points, “Disagree” scores 1 point, with higher scores indicating a higher level of career readiness. In the present study, the coefficient of internal consistency for the whole scale was 0.872. The sub-scales registered internal consistencies of 0.813 (Career Concern), 0.806 (Career Curiosity), and 0.811 (Career Confidence), with test–retest reliability ranging from 0.657 to 0.792. The PLS analysis demonstrated a factor loading larger than 0.50 for all items and the Average Variance Extracted (AVE) was 0.576. The CFA revealed the three-factor model provided an acceptable fit to the data (χ^2^/df = 3.12, CFI = 0.912, NFI = 0.911, RMSEA = 0.063).

The Vocational Identity Status Assessment (VISA) contains three sub-scales of career exploration, career commitment, and career reconsideration. The first two of these were selected for the current study. Career exploration and career commitment refer to the process by which students are committed to transitioning to the role of worker, with the corresponding scales each containing ten items. VISA used a 5-point Likert-type scale (1 = strongly disagree, 5 = strongly agree); higher scores indicate greater levels of career exploration and commitment. The internal consistency for the whole scale was 0.865 (0.825 for Career Exploration and 0.792 for Career Commitment) and the test–retest reliability was 0.645 and 0.627 for each subscale. The PLS demonstrated a factor loading above 0.50, with an AVE of 0.604. The CFA indicated that a two-factor model provided an acceptable fit to the data (χ^2^/df = 2.36, CFI = 0.932, NFI = 0.925, RMSEA = 0.054).

#### Career Decision-Making Difficulties

To measure participants’ career decision-making difficulties, we selected the Career Decision-Making Difficulties Questionnaire (CDDQ; Gati et al., 1996). It was divided into three sub-scales–Lack of Readiness, Lack of Information, and Inconsistent Information. The original form of CDDQ contained 34 items, 32 career decision-making difficulty items, and two validity items. CDDQ used a 9-point Likert-type scale (1 = does not describe me, 9 = describes me well); a higher score represents a higher level of career decision-making difficulties. In the current study, the coefficient of internal consistency for the entire scale was 0.812, for each sub-scale this was 0.627 (lack of Readiness), 0.952 (Lack of Information), and 0.920 (Inconsistent Information). The test–retest reliability ranged from 0.604 to 0.875. Results of PLS indicated a factor loading greater than 0.50 for all items and an AVE of 0.547. The results of the CFA indicated that the three-factor model provided an acceptable fit to the data (χ^2^/df = 3.37, CFI = 0.931, NFI = 0.922, RMSEA = 0.062).

### Procedure

The online career intervention “Preparing for My Future Selection” was developed and implemented during the Covid-19 pandemic while all Chinese schools were closed to face-to-face instruction. The online intervention was similar to the outline of traditional face-to-face career intervention but transited into an online one by using the internet and information technology tools. The intervention consisted of a series of career courses, a career assessment and guided access to a database providing information about university majors. The control group did not access the intervention or any other career interventions during the research period, but they participated in it after the study had been completed. The online career course focused on decision-making skills and strategies for academic or professional plans and comprised five sections: (1) *What is career planning*? This section aimed to raise participants’ awareness of career planning by detailing its importance for high school students and the factors to be considered when planning. (2) *Exploration of vocational interests*. This section explained the relationship between vocational interests and career planning. We taught participants about Holland’s six categories (Realistic, Investigative, Artistic, Social, Enterprising, Conventional) of interests (Spokane and Holland, 1995), and some approaches to clarify vocational interests (e.g., the formal approach of assessment, and informal approaches such as card-sorting activities related to the topic). (3) *Exploration of academic self-concept*. In this section, participants studied academic self-concept (Marsh et al., 2005) and its importance to planning their high school career. (4) *Subject selection*. In this section, we taught participants the relationship between subject selection and career planning. The gap between high school subjects and university majors is crucial to understand; for example, most majors in medicine require high school students to take science subjects rather than humanities. (5) *Exploration of majors*. In this section, we informed participants about university majors and the relationship between majors and vocations, helping them to narrow down their searches for suitable majors to study at university.

We also assigned students to use the online career assessment as homework. After section “Literature Review,” participants were invited to complete the Self-Directed Search–Chinese Version (Yang et al., 2006) to explore vocational interests; after section “Materials and Methods,” participants were invited to finish Self-Description Questionnaire II–Chinese Version (Leung et al., 2016) to explore academic self-concept. Upon completion, the students were provided with a report which interpreted their responses and suggested several potential university majors and occupations. Then, the students may further explore and consider what university majors to learn. Therefore, after sections “Results” and “Discussion,” participants were encouraged to use the university major database for searching more pivotal information about these potential university majors.

The online career course and career assessments extended students’ self-knowledge, while the database section increased their knowledge of potential university major options. Both thereby drew on aspects of the CIP pyramid of cognitive processing. Specifically, the online intervention addressed self-knowledge (see sections “Literature Review” and “Materials and Methods”; the career assessment for clarifying vocational interests and academic self-concept), occupational knowledge (see sections “Results” and “Discussion”; the database for gathering information about university majors); decision-making skills (see section “Introduction”), and considering about career decision-making process (see sections “Literature Review,” “Materials and Methods,” “Results,” and “Discussion”).

The online course was delivered by two instructors: Instructor A taught Section one, two, three, and Instructor B taught the remaining sections. The two instructors are university professors, majoring in career counseling and guidance. The intervention was a synchronous one, classes were taught every Monday evening for five consecutive weeks. Each section consisted of a 45-min live webcast; and the last 5 min of each section were available to students to submit typed questions, one or two of which were answered by instructors. The pre-test, using the three instruments described above, was completed before the first live webcast. The same scales completed 1 day after the last live webcast served as the post-test. Every participant had an account to log in the online platform and the platform collected the participation rate for each component (Section 1 = 99.70%, Section 2 = 96.34%, Section 3 = 96.20%, Section 4 = 95.12%, Section 5 = 94.05%; use of SDS = 96.27%, use of SDQ-II = 94.97%; use of the database = 92.53%). Participants also evaluated the intervention *via* a questionnaire. The results found that 91.21% of participants were satisfied with the teachers’ teaching competence, 87.24% considered that the career intervention had been helpful and 90.46% of participants approved the arrangement of the five sections. The study has been approved by the Research Ethics Committee of the Nanjing Normal University.

### Data Analysis

Data were analyzed by using SPSS 22.0, AMOS 16.0 and SmartPLS. A two-way MANOVA was used to examine the interaction effect between group (experimental group vs. control group) and time (pre- vs. post-test) in career readiness and CDDQ. A three-way MANOVA measured the moderation effect of gender on intervention effects (time × group × gender). The effect size was measured by partial eta-squared (η^2^P), which estimates the percentage of variances explained by the variables; the threshold values for the index η^2^P were 0.01, 0.06, and 0.14, respectively, representing a small, moderate and large effect size (Lachenbruch and Cohen, 1989).

## Results

### Descriptive Statistics and Correlation Analysis

Table 1 presents the summary of means and standard deviations of the pre-test and post-test among all the measurements for the experimental and control group and by gender. Table 2 presents the zero-order correlations of the pre-test and post-test among participants’ scores on CMI-Form C, VISA, and CDDQ.

**TABLE 1:** Means and standard deviation among the study variables.

	Group				Gender			
	Control (*n* = 356)		Experimental (*n* = 601)		Boys (*n* = 521)		Girls (*n* = 436)	
	*M*	*SD*	*M*	*SD*	*M*	*SD*	*M*	*SD*
**Career concern**								
Pre-test	1.80	0.19	1.81	0.19	1.80	0.19	1.83	0.17
Post-test	1.81	0.19	1.82	0.21	1.80	0.21	1.82	0.19
**Career curiosity**								
Pre-test	1.61	0.33	1.59	0.33	1.62	0.33	1.56	0.33
Post-test	1.60	0.33	1.73	0.31	1.68	0.33	1.67	0.32
**Career confidence**								
Pre-test	1.67	0.29	1.67	0.29	1.71	0.28	1.63	0.29
Post-test	1.68	0.31	1.76	0.29	1.73	0.29	1.72	0.30
**Career exploration**								
Pre-test	2.88	0.34	2.86	0.33	2.75	0.35	2.76	0.31
Post-test	2.89	0.35	3.11	0.35	2.81	0.37	2.87	0.36
**Career commitment**								
Pre-test	2.76	0.29	2.74	0.31	2.86	0.30	2.87	0.29
Post-test	2.77	0.32	2.81	0.30	3.04	0.31	3.02	0.31
**Lack of readiness**								
Pre-test	2.26	0.36	2.27	0.37	2.26	0.38	2.31	0.32
Post-test	2.22	0.38	2.24	0.43	2.21	0.43	2.26	0.39
**Lack of information**								
Pre-test	2.16	0.58	2.22	0.60	2.11	0.63	2.30	0.53
Post-test	2.17	0.63	2.04	0.65	2.04	0.67	2.14	0.61
**Inconsistent information**								
Pre-test	2.15	0.53	2.15	0.56	2.06	0.58	2.25	0.48
Post-test	2.14	0.57	2.02	0.64	2.02	0.64	2.12	0.58

**TABLE 2:** Zero-order correlations between the career maturity scale, career decision-making difficulties scale, and vocational identity status assessment.

	*Skewness*	*Kurtosis*	1	2	3	4	5	6	7	8
1 Career concern	–1.15	1.70	–	0.16***	0.15***	0.11**	–0.01	−0.20***	−0.13***	−0.13***
2 Career curiosity	–0.29	–1.13	0.29***	–	0.59***	0.21***	0.00	−0.47***	−0.62***	−0.52***
3 Career confidence	–0.56	–0.73	0.20***	0.62***	–	0.07*	−0.11*	−0.54***	−0.53***	−0.55***
4 Career exploration	–0.36	1.78	0.03	0.11***	0.07*	–	0.10*	–0.04	−0.22***	−0.13***
5 Career commitment	–0.29	1.71	−0.08*	–0.05	−0.09**	0.59***	–	0.10*	0.03	0.15***
6 Lack of readiness	0.31	0.23	−0.25***	−0.440***	−0.54***	0.01	0.17***	–	0.58***	0.58***
7 Lack of information	–0.34	–0.21	−0.21***	−0.50***	−0.57***	−0.14**	0.11**	0.70***	–	0.78***
8 Inconsistent information	–0.32	0.01	−0.20***	−0.50***	−0.59***	−0.09**	0.14**	0.71***	0.86***	–

***p < 0.001, **p < 0.01, and *p < 0.05.

*Values above the diagonal represent pretest correlations, and values below the diagonal represent post-test correlations.*

### Effects of the Intervention on Career Readiness

Table 3 presents the results of the two-way MANOVA with the effect sizes on career readiness. The results demonstrated that the effect of time (pre-test vs. post-test) was significant in career curiosity (*p* < 0.001) and career confidence (*p* < 0.001), but not in career concern (*p* > 0.05). The interaction effect of time by group was significant in career curiosity[*F*_(1,955)_ = 32.79, *p* < 0.001; Partial η^2^ = 0.033], and in career confidence [*F*_(1,955)_ = 7.32, *p* < 0.01; Partial η^2^ = 0.008]. This shows that the online career intervention significantly increased the experimental group members’ career curiosity and career confidence.

**TABLE 3:** The effects of the intervention on career readiness and the moderating effect of gender.

	F	*p*	Partial η^2^
**Career concern**			
Time	0.07	0.794	0.000
Time × Group	0.55	0.460	0.001
Gender × Group	1.06	0.303	0.001
Time × Group × Gender	0.35	0.553	0.000
**Career curiosity**			
Time	23.42	0.000	0.024
Time × Group	32.79	0.000	0.033
Gender × Group	11.76	0.001	0.012
Time × Group × Gender	2.20	0.138	0.002
**Career confidence**			
Time	16.34	0.000	0.017
Time × Group	7.32	0.007	0.008
Gender × Group	3.10	0.000	0.013
Time × Group × Gender	0.01	0.924	0.000
**Career exploration**			
Time	70.55	0.000	0.069
Time × Group	71.95	0.000	0.070
Gender × Group	30.07	0.000	0.031
Time × Group × Gender	0.09	0.771	0.000
**Career commitment**			
Time	5.68	0.017	0.006
Time × Group	4.47	0.035	0.005
Gender × Group	1.17	0.071	0.001
Time × Group × Gender	0.78	0.378	0.001

The pre-test and post-test differences were significant in career exploration (*p* < 0.001) and career commitment (*p* < 0.05). The interaction effect of time by group was significant in career exploration [*F*_(1,955)_ = 71.95, *p* < 0.001; Partial η^2^ = 0.070], meaning that the experimental group explored their career options more fully as a result of the intervention. The interaction effect of time × group was also significant in career commitment [*F*_(1,955)_ = 4.47, *p* < 0.05, Partial η^2^ = 0.005], indicating that students who were in the intervention group also displayed higher levels of career commitment.

### The Effect of the Intervention on Career Decision-Making Difficulties

Table 4 presents the results of repeated measures with the effect size for career decision-making difficulties. We found the career intervention had a significant effect in addressing lack of information (*p* < 0.001), inconsistent information (*p* < 0.01), and lack of readiness (*p* < 0.05). The interaction effect of time by group was significant in lack of information [*F*_(1,955)_ = 12.74, *p* < 0.001; Partial η^2^ = 0.013], and inconsistent information [*F*_(1,955)_ = 4.74, *p* < 0.001; Partial η^2^ = 0.014], but it was not significant in lack of readiness (*p* > 0.05). In other words, the intervention decreased the high school students’ difficulties in career decision-making as these pertained to lack of information and inconsistent information.

**TABLE 4:** The effects of the intervention on career decision-making difficulties and the moderating effect of gender.

	F	*p*	Partial η^2^
**Lack of readiness**			
Time	4.30	0.038	0.004
Time × Group	0.15	0.697	0.000
Gender × Group	0.67	0.412	0.001
Time × Group × Gender	1.27	0.260	0.001
**Lack of information**			
Time	12.60	0.027	0.003
Time × Group	12.47	0.000	0.013
Gender × Group	1.12	0.006	0.011
Time × Group × Gender	0.42	0.516	0.000
**Inconsistent information**			
Time	8.39	0.004	0.009
Time × Group	4.74	0.000	0.014
Gender × Group	1.38	0.009	0.009
Time × Group × Gender	1.73	0.188	0.002

### The Moderating Effect of Gender

The interaction effects of time by gender by group were not found in any variables (*p* > 0.05), which means no significant moderating effects of gender were detected. In other words, gender did not influence the impact of the intervention on high school students’ career readiness or decision-making difficulties. Hypothesis 3 was not confirmed.

## Discussion

Results indicated that the CIP-based online intervention increased high school students’ career readiness, as predicted by the 1st hypothesis. It decreased high school students’ career decision-making difficulties, as hypothesis 2 predicted. These findings are consistent with the previous researches (Lam and Santos, 2018; Babarović et al., 2020).

Regarding high school students’ career readiness, both time and the interaction of time and group had a significant effect on career curiosity, career confidence, career exploration and career commitment. It is therefore apparent that the intervention provided knowledge and skills which encouraged students to search for information about majors and vocations. The online career assessments and the university major database prompted high school students to develop their knowledge of self and the vocational world in order to specify a clear career path. Learning systematic methods of career planning boosted the decision-making process and commitment to achieving their career goals. However, the interaction effect of time and group had no significant effect on career concern. The concern scores in the pre-test were high for both the control and experimental groups. This may be because students are strongly encouraged to consider their futures and concentrate on educational and vocational approaches which will support career development (Hartung, 2013). In Chinese high schools, students are encouraged to focus on their future careers: related slogans are everywhere on campuses, for instance. As a result, the participants’ high levels of career concern were already evident before they received the intervention and were affected minimally by the intervention. Regarding career exploration, the partial eta-squared for the interaction effect of time and group was 0.070, which could be considered moderate.

In terms of high school students’ career decision-making difficulties, the main effect of time and the interaction effect of time and group were significant for the categories of lack of information and inconsistent information. However, there was a small effect for lack of information and inconsistent information. Perhaps because high school students in the experimental group had gathered a lot of career information in a short period, they were unable to distinguish whether the information was useful or not, making it appear inconsistent. The finding that lack of readiness was not affected by the intervention may have resulted from the instrument used to measure it. The Cronbach’s-alpha internal consistency for lack of readiness was 0.627, which was lower than other sub-scales. Other researchers have also found the Cronbach’s-alpha internal consistency for lack of readiness was weak (Rochat, 2019; Levin et al., 2020), bringing the validity of this construct into question.

Regarding gender, we found no moderating effect of gender on the online career intervention. Hypothesis 3 was not verified. Although some studies found gender had a significant impact on career intervention (Cassie and Chen, 2012; Hechtlinger and Gati, 2019), our finding was consistent with other studies (Nota et al., 2016; Thul-Sigler and Colozzi, 2019; Gu et al., 2020). The online career intervention was perceived to be the same appropriate by both girls and boys. Probably the information technology in the intervention is perceived to be proper by both male students and female students, and it offers the chance of involving adolescents in an interactive approach regardless of gender (Rainie, 2010).

Our findings indicate that the synchronous online interventions to help students concentrate on career development could lead to more productive behaviors and investment in their future (Pordelan and Hosseinian, 2020). Due to the limited interaction with teachers, student learning in online environments is necessarily more self-directed than its face-to-face equivalent. Online career interventions encourage students to work independently on related tasks and activities, developing their enthusiasm for learning. In summary, the online intervention we developed provided a series of resources enabling students to meet the challenges of choosing a career more effectively.

## Implications for Practice

Our study indicated that online career interventions have considerable potential to improve current approaches to the provision in Chinese high schools. The current shortage of specialized careers officers means that some staff are currently required to provide face-to-face support to multiple schools. This is both inefficient in terms of time and expense to teachers and students. It is also riskier during the current Covid-19 pandemic. In contrast, online career interventions can provide services that are inexpensive, efficient, and safe. While such interventions should not replace school counselors as primary teaching agents, they can undoubtedly make a vital contribution to fundamental career services (Cerrito et al., 2018).

Our findings, moreover, demonstrate the potential effectiveness of CIP theory-based interventions in enhancing students’ self-knowledge, knowledge of available careers options, and decision-making skills (Osborn et al., 2020). The contents of career interventions should be tailored to the Chinese context and should also address the developmental needs of students. For example, many schools currently overlook guidance on selecting subjects and most students base their selections on academic scores, ignoring complex factors such as interests, academic self-concept and personality altogether.

## Limitations and Future Research Design

Notwithstanding the valuable contribution we believe this study makes to current understandings of high school career services, there were several limitations that are worthy of mention. First, the participants in our study attended excellent high schools, were high achievers and were psychologically well-adjusted. Future research should recruit more diverse samples, such as those from lower socioeconomic groups or lower-achieving students. Second, as mentioned above, the instruments we used were not developed for the Chinese context: future research may benefit from developing scales in the local context to improve their fit to Chinese students.

In addition, the CIP approach to career intervention emphasizes the importance of assessing emotions and negative attitudes to careers (Bullock-Yowell et al., 2011), but these two elements were outside the scope of the present study, and it is therefore recommended that such emotional and/or attitudinal changes are evaluated in future research. Furthermore, the intervention lasted only 5 weeks and no follow-up was conducted after the intervention: a longitudinal study is required to determine the durability of the positive types of changes we observed. Finally, not every student was able to attend school during the study period owing to the pandemic: it will be interesting to compare the effects of online vs. traditional face-to-face career interventions in the future.

## Data Availability Statement

The original contributions presented in the study are included in the article/supplementary material, further inquiries can be directed to the corresponding author.

## Ethics Statement

This study involving human participants was reviewed and approved by the Ethics Board of Nanjing Normal University. The ethics committee waived the requirement of written informed consent for participation.

## Author Contributions

SC and XG contributed to the conception and design of the study. SC and HC conducted the statistical analyses and wrote the first draft of the manuscript. HL wrote several sections of the manuscript. All authors contributed to the manuscript revision, and read and approved the submitted version of the manuscript.

## Conflict of Interest

The authors declare that the research was conducted in the absence of any commercial or financial relationships that could be construed as a potential conflict of interest.

## Publisher’s Note

All claims expressed in this article are solely those of the authors and do not necessarily represent those of their affiliated organizations, or those of the publisher, the editors and the reviewers. Any product that may be evaluated in this article, or claim that may be made by its manufacturer, is not guaranteed or endorsed by the publisher.
